# Higher 90-Day Mortality after Surgery for Hip Fractures in Patients with COVID-19: A Case–Control Study from a Single Center in Italy

**DOI:** 10.3390/ijerph18105205

**Published:** 2021-05-13

**Authors:** Alberto Grassi, Luca Andriolo, Davide Golinelli, Dario Tedesco, Simona Rosa, Pasquale Gramegna, Jacopo Ciaffi, Riccardo Meliconi, Maria Paola Landini, Giuseppe Filardo, Maria Pia Fantini, Stefano Zaffagnini

**Affiliations:** 1 II Clinica Ortopedica e Traumatologica, IRCCS Istituto Ortopedico Rizzoli, Via Pupilli 1, 40136 Bologna, Italy; alberto.grassi@ior.it (A.G.); stefano.zaffagnini@ior.it (S.Z.); 2 Department of Biomedical and Neuromotor Sciences (DIBINEM), Alma Mater Studiorum—University of Bologna, Via San Giacomo 12, 40126 Bologna, Italy; davide.golinelli@unibo.it (D.G.); simona.rosa@unibo.it (S.R.); mariapia.fantini@unibo.it (M.P.F.); 3 IRCCS Istituto Ortopedico Rizzoli, Via Pupilli 1, 40136 Bologna, Italy; dario.tedesco@regione.emilia-romagna.it (D.T.); mariapaola.landini@ior.it (M.P.L.); 4 Medicina e Reumatologia, IRCCS Istituto Ortopedico Rizzoli, Via Pupilli 1, 40136 Bologna, Italy; pasquale.gramegna@ior.it (P.G.); jacopo.ciaffi@ior.it (J.C.); riccardo.meliconi@ior.it (R.M.); 5 Applied and Translational Research (ATR) Center, IRCCS Istituto Ortopedico Rizzoli, Via di Barbiano 1/10, 40136 Bologna, Italy; g.filardo@biomec.ior.it

**Keywords:** hip fracture, COVID-19, coronavirus, elderly

## Abstract

The mortality of hip fracture (HF) patients is increased by concomitant COVID-19; however, evidence is limited to only short follow-up. A retrospective matched case–control study was designed with the aim to report the 90-day mortality and determine the hazard ratio (HR) of concomitant HF and COVID-19 infection. Cases were patients hospitalized for HF and diagnosed with COVID-19. Controls were patients hospitalized for HF not meeting the criteria for COVID-19 diagnosis and were individually matched with each case through a case–control (1:3) matching algorithm. A total of 89 HF patients were treated during the study period, and 14 of them were diagnosed as COVID-19 positive (overall 15.7%). Patients’ demographic, clinical, and surgical characteristics were similar between case and control groups. At 90 days after surgery, 5 deaths were registered among the 14 COVID-19 cases (35.7%) and 4 among the 42 HF controls (9.5%). COVID-19-positive cases had a higher risk of mortality at 30 days (HR = 4.51; *p* = 0.0490) and 90 days (HR = 4.50; *p* = 0.025) with respect to controls. Patients with concomitant HF and COVID-19 exhibit high perioperative mortality, which reaches a plateau of nearly 30–35% after 30 to 45 days and is stable up to 90 days. The mortality risk is more than four-fold higher in patients with COVID-19.

## 1. Introduction

As of 7 May 2021, 156,151,468 confirmed cases and 3,258,414 deaths of coronavirus disease 2019 (COVID-19), an infectious disease caused by the severe acute respiratory syndrome—coronavirus 2 (SARS-CoV-2), have been reported worldwide [1]. Older individuals are overrepresented among COVID-19 deaths. Evidence shows that the infection fatality ratio is very low for people between the ages of 15 and 44, increasing to 3.1% for 65–74-year-old people and to 11.6% for older ages [2,3,4,5].

Hip fracture (HF) is among the clinical conditions that are frequent in elderly patients and that potentially expose them to higher mortality risk [6,7]. Patients that incur HF represent a particularly challenging patient population in this context [8,9,10], due to the high postoperative mortality rate caused by the morbidity of the surgical procedure, functional impairment, and limited mobility [6,7,11]. Overall, HF in elderly patients represents one of the most relevant concerns for orthopedic surgeons [7,12,13]. This event is usually burdened by a high mortality rate, estimated around 5–7% at 30 days [12] and nearly 25% at 1 year [13]. Several risk factors of increased mortality have been identified, such as older age, male sex, concomitant pathologies, or surgical timing [14].

In these challenging patients, COVID-19 has been recently suggested as an independent predictor of mortality after HF as well [15]. A recent meta-analysis of 28 studies, including 596 COVID-19-positive patients with HF, reported an overall mortality of 35%. However, as suggested by this review and other early reports, several biases can be found. In fact, most of the studies reported early intrahospital or 30-day mortality, with only one study exceeding this temporal landmark [16]. Moreover, the comparison of mortality between COVID-19 and non-COVID-19 patients was reported only in a subgroup of clinical studies, often without adjusting mortality for relevant confounding factors such as age, sex, or comorbidities [15]. Another relevant issue was the timing of COVID-19 and how this affected survival probability. Defining follow-up rules is crucial, given that following patients for 30 days after surgery or after COVID-19 diagnosis is not the same. Assuming the hypothesis that the survival rate after acquiring COVID-19 is the same, independently, if the infection happens perioperatively or 7, 14, or 21 days later, this would have a profound effect on the potential mortality rates evaluated at short-term follow-up after surgery [15]. This inconsistent practice in reporting follow-up after admission rather than after diagnosis of COVID-19 may result in a greater mortality rate in the COVID-19 group with longer follow-up [14]. In this regard, Clement et al. [15] highlighted the poor reporting on the length of follow-up, whether the patient was COVID-19 positive at admission, and the time at which COVID-19 was diagnosed following admission. Thus, the authors suggested minimum reporting criteria needed for studies that investigate the association of COVID-19 and mortality in HF patients, including patient demographic and comorbidities, length of follow-up, and time of COVID-19 diagnosis (at or after admission), as well as a minimum of 30 days follow-up after diagnosis of COVID-19 and, if possible, adjusted mortality rate/risk. Moreover, considering the different settings of National Health Systems in terms of COVID-19 management, access to medical care, promptness of HF treatment, and population demographics, the analysis of COVID-19 mortality in patients with HF of a specific country or region has an intrinsic relevance [17,18]. In fact, to date, most of these studies come from the UK or USA, especially those comparing the mortality rate among patients with or without COVID-19. In this regard, the analysis of the Italian scenario represents a relevant source of data regarding the medium-term trend of COVID-19 infection and mortality during the first wave of the pandemic. Moreover, the COVID-19 outbreak showed a tremendous impact on all orthopedic trauma activities throughout the Italian country, but HF showed a minor reduction [19].

Therefore, the present study aims to report and analyze the mortality of HF patients with COVID-19 during the first wave of the pandemic in a single Italian orthopedic center, according to the aforementioned minimum reporting criteria. To this purpose, patients with and without COVID-19 undergoing surgery for HF between March and May 2020 were followed up to 90 days from surgical intervention, hospital admission, and COVID-19 diagnosis. The hypothesis was that a progressive increase of mortality is present from 30 to 90 days in HF patients with COVID-19 and that their mortality is significantly higher than that of similar patients without COVID-19.

## 2. Materials and Methods

### 2.1. Study Design and Setting

A retrospective matched case–control study was designed to address the objective of this study. Cases and controls were identified among all consecutive patients aged ≥65 hospitalized for hip fracture at the Rizzoli Orthopedic Institute (Bologna, Italy) from 1 January 2019, to 31 May 2020. The Rizzoli Orthopedic Institute is a third-level monospecialty research hospital in Bologna, Italy, which is also a member of the International Society of Orthopaedic Centers (ISOC) [20]. Cases and controls were retrospectively identified in the hospital administrative and discharge records databases, with a ratio of 1:3. Patient characteristics, including the number and type of comorbidities, were extracted from the hospital discharge records database and medical charts. Access to all medical information regarding the included patients was granted to the authors of this study, which were among the clinicians that operated and managed the patients. Information on 7-, 14-, 30-, 45-, and 90-day mortality from the day of hospital admission, COVID-19 diagnosis, and surgery for HF were retrieved from the medical charts and regional mortality database.

All data were anonymous and used respectfully of any privacy and ethical issues. Ethical review board approval was obtained from the AVEC Ethics Committee on 04/06/2020 with Protocol Number 0007798.

### 2.2. Case Definition

A case was defined as a patient of any gender and nationality, aged ≥65, hospitalized for HF (including both medial and lateral femoral neck fractures) at the Rizzoli Orthopaedic Institute during the period March–May 2020, and with a diagnosis of COVID-19 confirmed by internal medicine physicians and based on either the clinical criteria of the Chinese Clinical Guidance for COVID-19 Pneumonia Diagnosis and Treatment [21] or with a SARS-CoV-2 positive result obtained through RT-PCR (reverse-transcription polymerase chain reaction)molecular test on a nasopharyngeal swab, as defined by the WHO guidelines [22].

### 2.3. Control Definition

Three controls were included for each case of HF with concomitant COVID-19. Controls were defined as patients hospitalized for HF at the Rizzoli Orthopedic Institute that did not result eligible for inclusion in the “case” group, either due to receiving a negative RT-PCR molecular test result on a nasopharyngeal swab or not meeting the clinical criteria for COVID-19 pneumonia [22]. Controls were individually matched with each case through a case–control (1:3) matching algorithm that accounted for gender, age (±3 years), medial or lateral femoral neck fracture, surgical intervention code (ICD-9-CM codes), and number of comorbidities (0–1 or ≥2 comorbidities), as defined by the Elixhauser Comorbidity Index (ECI) [23]. Case–control matching through the ECI was only based on the number of comorbidities (0–1 or ≥2 comorbidities). Overall, if there were more than 3 candidate controls for each case, the choice was made through random sampling. Both cases and controls were treated by the same orthopedic team composed of experienced orthopedic surgeons.

### 2.4. Statistical Analysis

Numerical variables were summarized as mean (standard deviation); categorical variables were summarized as frequencies and percentages. Patients’ characteristics and mortality data extracted both from medical charts and the regional mortality database were listed. We also described the patients’ comorbidity profile, identified from administrative databases through the ECI [23], and listed each patients’ comorbidities. We used the *t*-test for continuous variables and the chi-square or Fisher exact test for categorical variables.

We performed a Cox regression to calculate survival curves of cases at 7, 14, 30, 45, and 90 days from three starting points: hospital admission, surgery, and COVID-19 diagnosis. We performed a Cox regression to calculate 90-day survival curves and hazard ratios (HRs) of matched cases and controls. The HR is a quotient of hazards of two groups and states how much higher the death rate is in one group than in the other group. The HR is a descriptive measure used to compare the survival times of two different groups of patients and it should be interpreted as a relative risk [24]. Odds ratios at the same time points and the risk difference were calculated as well. The sample size was determined by the total number of patients affected by COVID-19 and HF according to inclusion and exclusion criteria, and a post hoc power analysis (calculated with Stata software, version 15.0—StataCorp LLC, College Station, TX, USA), based on the chi-square test, resulted in 61.6% power. The level of statistical significance was set at *p* < 0.05. Statistical analyses were performed using SPSS 14.0, version 14.0.1 (SPSS Inc., Chicago, IL, USA), and JMP, version 12.0.1 (SAS Institute Inc., Cary, NC, USA, 1989–2007).

## 3. Results

### 3.1. Patient Demographic

A total of 89 HF patients were treated during the study period, and 14 of them were diagnosed as COVID-19 positive; therefore, the overall COVID-19 prevalence in HF patients was 15.7%. Considering that 14 patients were included in the case group and matched each with 3 HF patients without COVID-19, a total of 42 patients were included in the control group.

Patients’ demographic, clinical, and surgical characteristics were similar between the two groups (Table 1), and no statistically significant differences were found; the mean age among COVID-19 positive cases and negative controls was 82.9 years and 83.1 years, respectively, while patient sex was predominantly female (92.9%). Fracture diagnosis was 50% femoral neck fracture and 50% intertrochanteric fracture in both case and control groups.

### 3.2. COVID-19 Patients Characteristics

Of the 14 HF patients diagnosed with COVID-19, only 1 was male, 10 (71%) were older than 80 years, and the remaining were between 70 and 79 years (29%). The most common comorbidities calculated with the ECI Index were cardiac arrhythmia (42.9%), chronic pulmonary disease (28.6%), dementia (21.4%), uncomplicated hypertension (21.4%), and congestive heart failure (14.3%) (Table 2). No statistically significant differences were found between cases and controls’ comorbidities.

The admission occurred the same day of the trauma in 50% of the cases, while in the other 50%, there was a 1-day delay. Surgery was performed within 48 h from the admission in 10 cases (71%), while in 4 cases surgery was delayed due to concomitant pathologies to address.

Based on symptoms and tests, 5 patients (36%) were COVID-19 positive at hospital admission, 4 (28%) were diagnosed within the first week, and 5 (36%) after 7 days. COVID-19 diagnosis was based on a positive test in 11 cases (76%) and based on clinical criteria in 3 cases (24%). Overall, six patients (43%) were asymptomatic during the hospital stay, but one of them became symptomatic and tested COVID-19 positive after hospital discharge (Table 3).

At 90-days after surgery, 5 deaths were registered among the 14 COVID-19 cases (35.7%). All were 87 years old or older, except for one male patient who was 79-year-old. Seven- and fourteen-day mortality calculated from hospital admission was lower than that calculated from COVID-19 diagnosis or surgery (Table 4).

**Figure 1:**
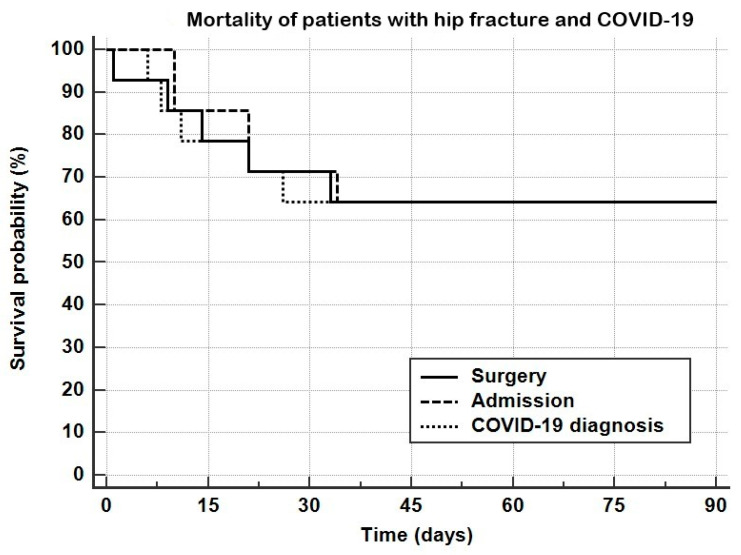
Ninety-day survival of COVID-19 patients calculated for time from surgery, time from hospital admission, and time from COVID-19 diagnosis.

However, the mortality rates overlap at the 45-day assessment (Figure 1).

### 3.3. Case–Control Comparison

A total of 4 deaths were reported out of 42 HF patients of the control group (9.5%). The risk difference (RD) between cases and controls was 26.2%. Overall, deceased patients were older than those alive, despite not statistically significant due to the limited number of patients (Table 5).

**Figure 2:**
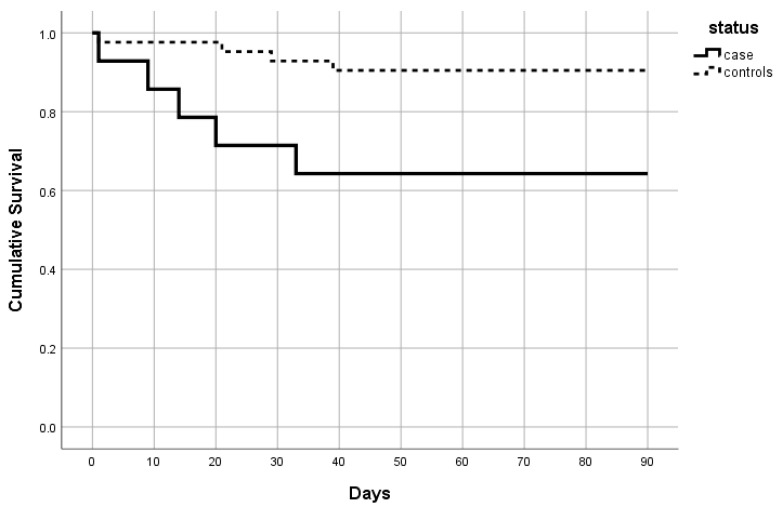
Ninety-day survival curve of cases and controls.

Figure 2 shows the 90-day survival curve of cases and control groups. The HRs for COVID-19-positive cases as compared to controls identified a higher risk of mortality in COVID-19 patients at 30 days (HR = 4.5; *p* = 0.0490) and up to 90 days (HR = 4.5; *p* = 0.025), with similar values. Significant ORs were found at 14 days and after 45 days (Table 4). The HRs and ORs were thus substantially stable from 30 to 90 days, after an initial peak at 14 days.

## 4. Discussion

Patients with HF and concomitant COVID-19 suffered a significantly higher risk of deaths compared to controls in this matched case–control study, despite having similar age, gender, number of comorbidities, and despite undergoing the same surgical procedure in the same setting and clinical center. The most important finding of the present study was that the risk of mortality for patients with HF and COVID-19 is nearly quadrupled at 30 days and maintained up to 90 days. So far, this represents the longest follow-up available for HF patients with COVID-19. Moreover, this analysis suggests that mortality rates could differ if the final endpoint is calculated from admission, surgery, or COVID-19 diagnosis, due to a relevant number of postoperative COVID-19 diagnoses. However, this issue seemed not to be relevant after 30 days, when mortality rates between cases and controls start to overlap.

The latter aspect, related to the timing of COVID-19 diagnosis and its effect on mortality rates, has been recently pointed out in the meta-analysis by Clement et al. [15]. The authors suggest that if COVID-19 is acquired 7, 14, or 21 days after admission (or surgery), this would have a profound effect on the mortality rate, suggesting a potential underestimation in the current published reports. Notably, in the present study, mortality appeared lower if the final endpoint was calculated from admission as compared to COVID-19 diagnosis. On the other side, it is not unusual that COVID-19 diagnosis is delayed due to the hospital setting and test availability (at least during the “first wave”), and therefore, it should be clarified whether the timing of COVID-19 diagnosis is based on the onset of symptoms or the timing of the test to increase the precision of mortality rate calculation. Another issue to consider is that, due to the clinical and logistical complexity of COVID-19 patients [25,26], surgery could be delayed in the case of HF, thus resulting in a non-neglectable discrepancy between admission and surgery [27]. On the other hand, the potential bias in mortality calculation related to the aforementioned issues, together with those suggested by Clement et al. [15], seems valid only in the early perioperative period, as after the first month, no difference in mortality from admission, surgery, or COVID-19 diagnosis were noted in the present study. This seems straightforward because it is plausible that death in COVID-19 patients, independently from the presence of HF, would occur at least within 90 or even 60 days from its diagnosis [28]. Thus, given that the follow-up is longer than 30 days, such as in the present study, it appears to be irrelevant from which time point the final endpoint is calculated. Of course, deaths could be expected even after 90 days from surgery, but these should be imputed to the natural consequence of the HF itself rather than merely COVID-19 related.

Apart from methodological considerations, the data presented in this study offer several important clinical insights regarding HF and COVID-19. First of all, the 30-day mortality in this single Italian orthopedic hospital was 28.6%, which increased to 35.7% at 45 days and was stable up to 90 days. These rates substantially overlap with the crude unadjusted mortality rate of 35% reported in a meta-analysis of 596 patients with HF and COVID-19. However, the present study exceeds the follow-ups described in the meta-analysis and in similar reports on HF in the Italian scenario [29], as it is currently the report on COVID-19 and HF with the longest follow-up. Thus, based on the available data, it could be suggested that a mortality peak is present within the first 2 weeks, as demonstrated by the significant odds ratio with respect to patients without COVID-19; however, after nearly 30–45 days, the mortality rate reaches a plateau, which is almost stable up to 90 days. Therefore, a neglectable number of deaths should be expected after the first postoperative month.

Another strength of the present study, rarely found in the available published series, was the statistical matching of HF patients with COVID-19 to a control group of HF patients without COVID-19. By matching patients based on age, sex, comorbidities, and type of fractures, it was possible to control most of the confounding factors possibly responsible for affecting the mortality rate [15,16,30]. According to this analysis, significant HR of 4.5 and OR of 5.2 were found from 30 days and beyond. The odds ratio found in this study was lower than the weighted value of 7.1 reported in the meta-analysis by Clement et al. [15]. This could be due because almost all the studies included by the authors reported only crude mortality without any statistical adjustments. Only two studies adjusted the mortality for confounding factors [16,31]: Clement et al., in a multicentric UK study on 68 HF patients with COVID-19 and 1659 controls highlighted higher mortality in infected patients than in controls reporting a hazard ratio of 1.9 [16], while Hall et al., in another study including 27 patients and 290 controls, reported a HR of 3.5. Our data (HR = 4.5) are more consistent with the study of Hall et al., also due to the similarities in the study design [31]. Moreover, the more than double HR respect to Clement et al. could be due to their decision to include elective procedures and other traumas in the control group, thus dissipating the final cumulative risk within the two significant independent variables (HF and COVID-19) [15]. Therefore, it could be suggested that HF patients with COVID-19 have a nearly four-fold risk of death than HF patients without the infection.

Similarities can be found between the included patients and the worldwide patient characteristics belonging to the “first wave” of COVID-19. The mean age of surgery of 82.9 years was similar to what was reported in most of the available reports [15]. The only outlier was the average age of 71.9 years of the series by Kayani et al. [31]. The reported prevalence of 15.7% in the present study is similar to the 13% obtained from a recent meta-analysis [15], as well as the proportion of patients with COVID-19 at admission (36% present study vs. 35% meta-analysis [15]). These findings all suggest a similar behavior and similar effects of COVID-19 on patients with HF across different countries, or at least in those investigated up to now (mostly Europe and the New York City area [32]).

Finally, it is worthy to contextualize the results of the present study also in its specific population-related environment. The metropolitan city of Bologna (1,019,875 citizens as of 1 January 2020 [33]) is located in one of the most affected regions of Italy and Europe during the “first wave.” Before the pandemic, in the same metropolitan area, the 6-month mortality rate for HF among elderly patients was 16.4% [34]. This rate seems in trend with the 3-month mortality rate of 9.5% of patients with HF and no COVID-19 infection, despite minor variations that could be present due to the patient-matching process. Thus, when calculating the risk difference between mortality of COVID-19 and non-COVID-19 patients, the rate of 26.2% appears similar to the overall fatality rate of 24.3% described for female patients aged between 80 and 89 years in Italy during the “first wave” [35], which is a demographic profile similar to the present study. Accordingly, further studies should be aimed at identifying if the HF itself represents a risk factor of increased mortality in patients with COVID-19, as demonstrated for obesity, neoplastic diseases, and diabetes [36,37,38,39], as the opposite situation (COVID-19 as a risk factor for HF patients) seems a piece of consolidated evidence [16,32,40].

The present study has several limitations, which are, however, dictated by the extraordinary circumstances of the “first wave” of the COVID-19 pandemic, rather than by the authors’ responsibility or methodological shortcomings. The most important is the limited number of included patients with HF and COVID-19 diagnosis. Nevertheless, all patients with HF and COVID-19 treated in the hospital during the first wave were included in this study, with no exclusion. Moreover, the 14-patient series of the Rizzoli Orthopedic Institute represents one of the largest populations when considering monocentric studies. As an example, the largest available populations of 82 patients [30], 68 patients [16], 27 patients [31], and 23 patients [41] all derived from multicentric studies including 9, 9, 6, and 13 hospitals, respectively, thus accounting on average less than 10 patients for each hospital. Granted, performing a monocentric study did not allow us to collect a satisfactory number of patients to perform sophisticated statistical subanalyses. This did, however, allow us to have direct access to patient data, treat them personally [42], and allow us to perform an accurate data collection and analysis of patients and controls treated in the same setting. Moreover, the standard of care and COVID-19 diagnosis could differ among various centers, despite geographical proximity, thus resulting in spurious and heterogeneous cohorts. Another limitation of this study is that case–control matching for the variables included does not avoid other potential confounders not considered in the analyses or not available to the authors. A strength of the study was that COVID-19 diagnosis and management was led by a single clinician, allowing us to include in our series also patients with a “clinical” COVID-19 diagnosis even with a negative test. In a multicentric scenario with standardized test-based inclusion criteria, these patients would have been lost and included in the control group, unbalancing the final mortality estimation. Finally, being a monospecialty orthopedic center allowed us to identify a control group of HF patients by matching the cases taking into account the most relevant demographic and clinical characteristics. Thus, the minimum reporting standards of outcomes associated with COVID-19 suggested by Clement et al. [15] were fulfilled, strengthening the findings on the higher 90-day mortality after surgery for HF in patients with concomitant COVID-19.

## 5. Conclusions

The prevalence of COVID-19 among HF patients in a single Italian hospital was nearly 15%, with most of the patients admitted with a pre-existing COVID-19 or a COVID-19 diagnosis in the first week. Patients with concomitant HF and COVID-19 exhibit high perioperative mortality, which reaches a plateau of nearly 30–35% after 30 to 45 days and is stable up to 90 days. The mortality risk is nearly four-fold higher than that of HF patients without COVID-19.

## Figures and Tables

**Table 1:** Characteristics of cases and controls.

Variables Types	Variables	COVID-19 (*n* = 14)	Non-COVID-19 (*n* = 42)	*p*-Value
Matching variables	Sex; *n* (%)	13 (93%), F	39 (93%), F	>0.05
		1 (7%), M	3 (7%), M	
	Age; mean (SD), year	82.9 (7.1)	83.1 (6.6)	>0.05
	ECI; *n* (%), comorbidities	5 (36%), 0–1	15 (36%), 0–1	>0.05
		9 (64%), ≥2	27 (64%), ≥2	
	ICD-9-CM; *n* (%), code	1 (7%), 79.15	3 (7%), 79.15	>0.05
		6 (43%), 79.35	18 (43%), 79.35	
		2 (14%), 81.51	6 (4%), 81.51	
		5 (36%), 81.52	15 (36%), 81.52	
Descriptive variables	Diagnosis; *n* (%)	7 (50%) femoral neck fractures	21 (50%) femoral neck fractures	1.000
		7 (50%) intertrochanteric fractures	21 (50%) intertrochanteric fractures	
	Procedure; *n* (%)	5 (36%) hip endoprosthesis	14 (33%) hip endoprosthesis	0.856
		6 (43%) intramedullary femoral nail	20 (47%) intramedullary femoral nail	
		2 (14%) THA	7 (17%) THA	
		1 (7%) ORIF	1 (3%) ORIF	
	Surgery time; mean (SD), minutes	67.6 (25.0)	69.0 (20.2)	0.844
	ASA; *n* (%)	12 (86%) ASA 3	37 (88%) ASA 3	0.943
		1 (7%) ASA 4	2 (5%) ASA 4	
		1 (7%) ASA 2	3 (7%) ASA 2	
	Fracture–surgery interval; mean (SD), days	3.2 (2.4)	2.8 (2.4)	0.584
	Admission–surgery interval; mean (SD), days	2.7 (2.6)	1.9 (1.5)	0.168
	Smokers; *n* (%)	4 (29%)	9 (21%)	0.415

F: female; M: male; ECI: Elixhauser Comorbidity Index; ASA: American Society of Anesthesiologists; THA: total hip arthroplasty; ORIF: open reduction and internal fixation; ICD-9-CM: International Classification of Diseases, Ninth Revision, Clinical Modification.

**Table 2:** Type of comorbidities (as defined by the Elixhauser Comorbidity Index) for cases and controls.

Main Comorbidity	COVID-19 (*n* = 14)		Non-COVID-19 (*n* = 42)		*p*-Value
	*n*	%	*n*	%	
Cardiac arrhythmia	6	42.9%	8	19.0%	0.080
Chronic pulmonary disease	4	28.6%	3	7.1%	0.058
Hypertension uncomplicated	3	21.4%	16	38.1%	0.210
Dementia	3	21.4%	11	26.2%	0.512
Congestive heart failure	2	14.3%	6	14.3%	0.651
Solid tumor without metastasis	1	7.1%	7	16.7%	0.349
Diabetes uncomplicated	1	7.1%	4	9.5%	0.633
Depression	1	7.1%	4	9.5%	0.633
Hypothyroidism	1	7.1%	3	7.1%	0.695
Fluid and electrolyte disorders	1	7.1%	2	4.8%	0.586
Diabetes complicated	1	7.1%	0	0.0%	0.250
Liver disease	1	7.1%	0	0.0%	0.250
Deficiency anemia	1	7.1%	0	0.0%	0.250
Other neurological disorders	0	0.0%	6	14.3%	0.162
Valvular disease	0	0.0%	5	11.9%	0.223
Renal failure	0	0.0%	3	7.1%	0.414
Blood loss anemia	0	0.0%	3	7.1%	0.414
Hypertension complicated	0	0.0%	3	7.1%	0.414
Metastatic cancer	0	0.0%	1	2.4%	0.750
Rheumatoid arthritis/collagen	0	0.0%	1	2.4%	0.750
Obesity	0	0.0%	1	2.4%	0.750
Peripheral vascular disorders	0	0.0%	1	2.4%	0.750

**Table 3:** Characteristics of COVID-19 patients.

Patient	ASA	Comorbidities	Orthopedic Diagnosis and Treatment	Admission–Surgery Interval	Length of Hospitalization	COVID-19 Diagnosis	Timing of Diagnosis	Clinical Symptoms	Laboratory Test Alterations	ABG Results	HRCT	Treatment	Exitus
F, 90 y	II	HT, CHD, K	Femoral neck fracture Hip endoprosthesis	1 day	9	Positive RT-PCR molecular test	Admission	Fever for 5 days	LC: Y; LP: N; LyP: N; PLT: 227; C: 0.73; IL-6: NA; LDH: NA; PCR: 15	NA	NEG	O2, LMWH, Antibiotic	no
F, 81 y	III	HT, COPD, D	Femoral neck fracture THA	1 day	22	Positive RT-PCR molecular test	22 days	Asymptomatic	LC: Y; LP: N; LyP: N; PLT: 400; C: 1.6; IL-6: NA; LDH: NA; PCR: 1.6	NA	NEG	LMWH, Antibiotic	no
F, 90 y	II	HT, DM, CHD	Intertrochanteric fracture Femoral intramedullary nail	1 day	10	Positive RT-PCR molecular test	11 days	Asymptomatic	LC: N; LP: N; LyP: N; PLT: 265; C: 0.58; IL-6: NA; LDH: NA; PCR: 5.9	SaO2: 93; PaO2: 52; PaCO2: 34; PF: 267	NEG	LMWH	no
F, 73 y	III	HT, COPD, K	Femoral neck fracture THA	2 days	8	Positive RT-PCR molecular test	Admission	Fever for 3 days Cough Desaturation	LC: Y; LP: N; LyP: Y; PLT: 248; C: 0.74; IL-6: NA; LDH: 336; PCR: 3.7	SaO2: 96; PaO2: 60; PaCO2: 33; PF: 286	NEG	O2, CPAP, HCQ, LMWH	no
F, 70 y	III	K	Intertrochanteric fracture Femoral intramedullary nail	2 days	11	Clinical criteria	2 days	Desaturation	LC: Y; LP: N; LyP: N; PLT: 420; C: 0.53; IL-6: NA; LDH: 232; PCR: 1.6	SaO2: 91; PaO2: 80; PaCO2: 23; PF: 857	POS	O2, HCQ, AZA, LMWH, Antibiotic	no
F, 72 y	III	HT, COPD	Femoral neck fracture Hip endoprosthesis	8 days	14	Positive RT-PCR molecular test	11 days	Asymptomatic	LC: N; LP: N; LyP: N; PLT: 87; C: 1.27; IL-6: NA; LDH: 228; PCR: 24	NA	NEG	LMWH, Antibiotic	no
F, 86 y	IV	HT, D, K	Intertrochanteric fracture Femoral intramedullary nail	3 days	8	Positive RT-PCR molecular test	Admission	Fever Cough	LC: N; LP: N; LyP: Y; PLT: 260; C: 0.85; IL-6: 66.6; LDH: NA; PCR: 4.6	SaO2: 98; PaO2: 69; PaCO2: 32; PF: 328	POS	O2, HCQ, AZA, LMWH, Antibiotic	no
F, 80 y	III	HT, S, COPD, D	Femoral neck fracture Hip endoprosthesis	2 days	11	Positive RT-PCR molecular test	2 days	Asymptomatic	LC: N; LP: N; LyP: N; PLT: 123; C: 0.89; IL-6: NA; LDH: NA; PCR: 5.6	SaO2: 91; PaO2: 68; PaCO2: 45; PF: 283	NEG	O2, HCQ, LMWH	no
F, 86 y	III	HT	Intertrochanteric fracture Femoral intramedullary nail	1 day	8	Positive RT-PCR molecular test	Admission	Asymptomatic	LC: N; LP: N; LyP: N; PLT: 278; C: 0.64; IL-6: NA; LDH: NA; PCR: NA	SaO2: 98; PaO2: 94; PaCO2: 34; PF: 448	NEG	HCQ, LMWH	no
F, 90 y	IV	HT, COPD, D, K	Femoral neck fracture Hip endoprosthesis	7 days	10	Positive RT-PCR molecular test	Admission	Cough	LC: N; LP: N; LyP: Y; PLT: 368; C: 0.53; IL-6: NA; LDH: NA; PCR: NA	SaO2: 98; PaO2: 71;PaCO2: 44; PF: 371	NEG	O2, LMWH	Yes (14-day postop)
F, 89 y	III	DM, CHD, COPD, D	Intertrochanteric fracture Femoral intramedullary nail	1 day	7	Positive RT-PCR molecular test	8 days	Asymptomatic	LC: N; LP: N; LyP: N; PLT: 272; C: 0.73; IL-6: NA; LDH: NA; PCR: 5.6	NA	NEG	LMWH	Yes (33-day postop)
F, 87 y	IV	CHD, COPD, D	Intertrochanteric fracture Femoral intramedullary nail	9 days	10	Clinical criteria	2 days	Fever for 3 days	LC: Y; LP: N; LyP: Y; PLT: 189; C: 0.69; IL-6: NA; LDH: 379; PCR: 26	SaO2: 83; PaO2: 48; PaCO2: 68; PF: 229	POS	O2, CPAP, HCQ, AZA, LMWH, Antibiotic	Yes (1-day postop)
F, 88 y	III	HT, DM, CHD, D, K	Femoral neck fracture Hip endoprosthesis	1 day	10	Positive RT-PCR molecular test	4 days	Dyspnea for 6 days	LC: N; LP: N; LyP: Y; PLT: 186; C: 1.20; IL-6: NA; LDH: NA; PCR: 8	SaO2: 86; PaO2: 44; PaCO2: 31; PF: 210	NEG	O2, LMWH	Yes (9-day postop)
M, 79 y	III	COPD, D	Femoral neck fracture ORIF	0 days	21	Clinical criteria	10 days	Fever for 11 days Dyspnea Desaturation	LC: Y; LP: N; LyP: Y; PLT: 300; C: 1.6; IL-6: 78; LDH: NA; PCR: 40	SaO2: 80; PaO2: 53; PaCO2: 31; PF: 252	POS	O2, CPAP, LMWH, Antibiotic	Yes (21-day postop)

F: female; M: male; y: years; HT: hypertension; DM: diabetes mellitus; CHD: coronary heart disease; COPD: chronic obstructive pulmonary disease; S: stroke; D: dementia; K: cancer; ORIF: open reduction and internal fixation; LC: leukocytosis; LP: leukopenia; LyP: lymphopenia; PLT: platelet count; C: creatinine; LDH: lactate dehydrogenase; CPR: C-reactive protein; NA: not assessed; ABG: arterial blood gas test; SaO2: oxygen saturation; PaO2: arterial partial pressure of oxygen; PaCO2: arterial partial pressure of CO2; PF: ratio of partial pressure arterial oxygen and fraction of inspired oxygen; O2: oxygen inhalation; CPAP: continuous positive airway pressure; HCQ: hydroxychloroquine; AZA: azathioprine; LMWH: low-molecular-weight heparin.

**Table 4:** Mortality rates and risk measures (hazard ratios and odds ratios between cases and controls) at 7, 14, 30, 45, and 90 days from hospital admission, surgery, and diagnosis of COVID-19.

Time Points and Outcome Measures		Mortality Rates and Risk Measures				
		7-Day	14-Day	30-Day	45-Day	90-Day
Mortality rate	COVID-19 Positive					
	Time from Admission	0.0%	14.3%	28.6%	35.7%	35.7%
	Time from Surgery	7.1%	21.4%	28.6%	35.7%	35.7%
	Time from COVID-19 Diagnosis	7.1%	21.4%	35.7%	35.7%	35.7%
	Control Group					
	Admission	2.4%	2.4%	7.1%	9.5%	9.5%
	Surgery	2.4%	2.4%	7.1%	9.5%	9.5%
		**7-day**	**14-day**	**30-day**	**45-day**	**90-day**
Risk Measures	Hazard Ratios					
	Value	3.1	9.6	4.5	4.5	4.5
	95% CI	0.12–78.0	0.9–95.6	1.0–20.2	1.2–16.7	1.2–16.7
	*p*-Value	=0.3972	=0.0766	=0.0490 *	=0.0250 *	=0.0250 *
	Odds Ratios					
	Value	3.1	11.2	5.2	5.2	5.2
	95% CI	0.2–54.0	1.1–119.3	1.0–27.1	1.2–23.7	1.2–23.7
	*p*-Value	=0.4281	=0.0449 *	=0.0502	=0.0300 *	=0.0300 *

* significant *p*-value (*p* < 0.05).

**Table 5:** Ninety-day survival of cases and controls and patients’ characteristics stratified for 90-day status (alive or deceased). No statistically significant differences have been found.

90-Day Survival	Patients’ Characteristics	COVID-19 Cases (*n* = 14)	Controls (*n* = 42)
Alive (%)		9 (64.3)	38 (90.5)
	Mean age (SD)	80.8 (7.7)	82.5 (6.6)
	Female (%)	9 (100)	35 (92.1)
	Mean ECI (SD)	1.7 (1.5)	2.1 (1.9)
Deceased (%)		5 (35.7)	4 (9.5)
	Mean age (±SD)	86.6 (4.4)	89.0 (1.4)
	Female (%)	4 (80)	4 (100)
	Mean ECI (SD)	2.2 (1.1)	1.5 (1.0)

ECI: Elixhauser Comorbidity Index.

## Data Availability

The data presented in this study are available on request from the corresponding author. The data are not publicly available due to ethical issues.
